# Misdiagnosis of a patient with congenital dysfibrinogenemia: A case report and literature review

**DOI:** 10.1002/jcla.24624

**Published:** 2022-08-10

**Authors:** Xinyan Chen, Jie Yan, Liqun Xiang, Faquan Lin

**Affiliations:** ^1^ Department of Clinical Laboratory The First Affiliated Hospital of Guangxi Medical University Nanning China

**Keywords:** congenital dysfibrinogenemia, diagnostic scheme, hypofibrinogenemia, misdiagnosis, pregnancy

## Abstract

**Background:**

We reported a patient with congenital dysfibrinogenemia who was misdiagnosed and reviewed relevant literature, in order to discuss the methods to reduce misdiagnosis.

**Methods:**

A 23‐year‐old pregnant woman was found to be with low fibrinogen in antenatal examination at another province teaching hospital, who was misdiagnosed to have hypofibrinogenemia. Fibrinogen infusion or cryoprecipitation was recommended if necessary. The patient came to our hospital for further diagnosis and treatment considering the safety of herself and the fetus. We examined the coagulation function and gene sequencing of the pregnant woman and her family members.

**Results:**

Fibrinogen (Clauss method) was significantly reduced in the patient and her mother, while the level of fibrinogen (PT‐derived method) was normal. Thrombin time was prolonged. Heterozygous mutation site was found in exon 2 of the FGA gene, c.104G > A(p.Arg35His).

**Conclusion:**

When the fibrinogen (Clauss method) is significantly reduced and the thrombin time is prolonged, PT‐derived method and the investigation of family coagulation function should be added, which can be used to diagnose and distinguish congenital dysfibrinogenemia from hypofibrinogenemia.

## Graphical abstract

Use PT‐derived assay when fibrinogen level is reduced and thrombin time is prolonged.

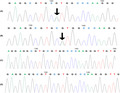

## 1 INTRODUCTION

Fibrinogen deficiencies can be divided into two types: Class I presents with abnormal fibrinogen quantity, including hypofibrinogenemia and afibrinogenemia, and Class II presents with abnormal fibrinogen quality, including dysfibrinogenemia and hypofibrinogenemia.^1^, ^2^ The World Bleeding Disorder Report 2017 issued by the World Federation of Hemophilia (http://www1.wfh.org/publications/files/pdf‐1714.pdf) indicated that fibrinogen deficiencies (including Class I and II defects) account for 6% of hemorrhagic disorders, except for hemophilia A, hemophilia B, and von Willebrand disease in 113 countries, but accounted for 37% of cases in China. Congenital dysfibrinogenemia (CD) is an inherited disease caused by defects in fibrinogen genes, leading to abnormal fibrinogen molecular structure and function.^3^, ^4^ CD is a class II fibrinogen disorder with an autosomal dominant inheritance pattern. Only one parent carrying the defective gene to the offspring can cause the disease, and the CD incidence rate is higher than that of hereditary afibrinogenemia. The clinical manifestations of CD are significantly heterogeneous, with 25% of patients presenting with bleeding symptoms, 20% with thrombotic diseases, and 55% without any clinical manifestations.^5^ The current methods for automatic fibrinogen detection are the Clauss method and PT‐derived fibrinogen assay. CD is prone to misdiagnosis or missed diagnosis if only one method is used to detect fibrinogen levels. Herein, we report a case of CD misdiagnosis and review the relevant literature to discuss a method that can reduce misdiagnosis or missed diagnosis.

## 2 PATIENTS AND METHODS

### 2.1 Patient

A 23‐year‐old pregnant woman had an induced abortion. In the 14th week of the second pregnancy (March 24, 2020), the obstetric examination at the Souzhu Hospital Affiliated to the M Medical University in other provinces revealed that her fibrinogen level was significantly reduced. Her vital signs were stable, and she presented with no signs of vaginal bleeding in the first trimester. Thromboelastography, routine blood examination, and genetic testing for thalassemia revealed no abnormalities. Fetal ultrasound measurements showed that the baby had grown well. The patient had been previously healthy. She and her family members had no history of unexplained bleeding and denied a family history of a consanguineous marriage. An expert consultation was held by nine experts from the departments of obstetrics and gynecology, laboratory, pediatrics, rheumatology, and immunology in Souzhu hospital. She was then diagnosed with hypofibrinogen and was considered to have an increased risk of bleeding or thrombosis that might endanger her life during pregnancy or delivery. Fibrinogen or cryoprecipitate infusion was recommended, if necessary. The patient complained that after being diagnosed with hypofibrinogenemia, she had trouble sleeping and eating, became depressed, and was often worried about the safety of herself and her fetus. During this period, she visited another provincial maternal and child hospital for diagnosis and treatment, but no definite diagnosis was made. On July 7, 2020, the patient presented to our hospital for further diagnosis and treatment.

### 2.2 Laboratory examinations

**FIGURE 1:**
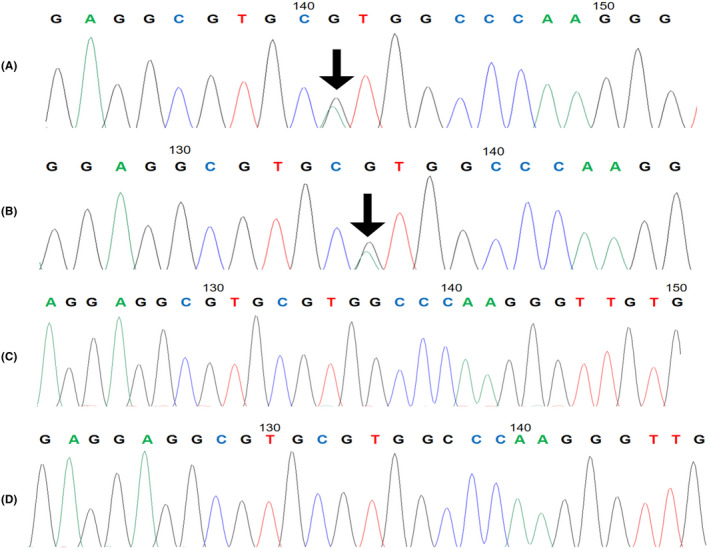
Fibrinogen gene sequencing results. (A) the proband; (B) the proband's mother; (C) the proband's father; (D) the proband's sister. The arrows indicate the mutation sites, both of which are heterozygous mutations in exon 2 of the FGA gene, c.104G > A (p.Arg35His).

Written informed consent was obtained from the patient and her family members. Ethical approval was obtained from the Medical Ethics Committee of the First Affiliated Hospital of Guangxi Medical University (approval no. 2015‐KY‐Guoji‐115) on 3/3/2015. Peripheral venous blood samples were collected to test liver, kidney, and coagulation functions of the patient and her family members. They had normal liver and kidney function. Four blood coagulation parameters were assessed. The thrombin time of the patient and her mother was prolonged, and the fibrinogen level (measured by the Clauss method) was significantly decreased; however, the fibrinogen level (measured by PT‐derived fibrinogen assay) was within the normal range. There were no abnormalities in the coagulation function of her sister or father (Table 1). Whole blood DNA was extracted from the patient and her family members, and the FGA, FGB, and FGG genes were amplified and sequenced to determine the mutation sites. DNA sequencing results showed that the patient and her mother had a heterozygous mutation c.104G > A (p.Arg35His) in exon 2 of the FGA gene. This mutation was not detected in other family members. Fibrinogen gene sequencing results for this family are shown in Figure 1.

**TABLE 1:** Coagulation tests of the proband and her family members

	Age (y)	APTT(s)	PT(s)	TT(s)	Fg^a^ (g/L)	Fg^b^ (g/L)
Proband	23	30.3	11.9	20.3	0.41	4.09
Mother	54	30.2	13.9	31.0	0.58	3.17
Father	55	29.6	11.8	15.0	2.72	3.28
Sister	28	34.5	10.8	10.5	2.52	2.97
Reference interval		23.0–40.0	9.0–15.0	9.0–15.0	2.00–5.00	2.00–5.00

Abbreviations: APTT, activated partial thromboplastin time; Fg, fibrinogen; PT, prothrombin time; TT, thrombin time.

^a^ Clauss method;

^b^ PT algorithms.

## 3 RESULTS

Based on coagulation function and gene sequencing results, the patient (proband) and her mother were diagnosed with CD. The patient and her family members had no history of bleeding or thrombosis. She showed normal results on thromboelastography, with no need for special treatments, such as fibrinogen infusion, during pregnancy, and before delivery. The patient presented with no abnormalities during pregnancy, and she delivered a full‐term baby naturally in our hospital on September 13, 2020. The delivery process went well. Soft birth canal examination showed the cervix was intact. The posterior vaginal wall was naturally lacerated and approximately 1 cm long, followed by a routine suture. There was less blood loss, approximately 110 ml within 2 h after delivery. The patient was discharged on the third day after the delivery.

## 4 DISCUSSION

Congenital dysfibrinogenemia diagnosis and differential diagnosis mainly rely on laboratory tests, and fibrinogen is a key diagnostic indicator. Among the Clauss method and PT‐derived assay currently used for fibrinogen detection of automatic coagulation analyzer, only the former method (the current preferred method) is used in most clinical laboratories to detect fibrinogen. The Clauss method is a thrombin method in which a sufficient amount of thrombin is added to the tested plasma for coagulation. The coagulation time is negatively correlated with the fibrinogen level. The fibrinogen level can be identified from the standard curve determined using the international standard fibrinogen reference plasma. The PT‐derived fibrinogen assay is used to determine fibrinogen level based on the absorbance difference of the vertical axis of the PT response curve. When prothrombin time is determined, all fibrinogen is converted to fibrin, and the amount of turbidity is proportional to the fibrinogen level (without the need for thrombin). Fibrinogen level can be calculated using the endpoint or rate method. Rumley et al.^6^ selected a random sample of the general population aged 24–64 years (*n* = 1373 of both sexes) for fibrinogen detection. A similar fibrinogen level was detected by the Clauss method and PT‐derived assay, which was 3.15 ± 0.96 and 3.20 ± 0.80 g/L, respectively. In a study by Miesbach et al.,^7^ two coagulation instruments from different manufacturers were simultaneously used to detect fibrinogen in 27 patients with CD. The median values of fibrinogen were 0.40 and 0.60 g/L by the Clauss method and 2.41 and 2.64 g/L by the PT‐derived assay, indicating significant differences in the detection results of the two methods. We previously enrolled 81 healthy controls and 73 patients with CD to study the clinical value of the Clauss method combined with the PT‐derived fibrinogen assay.^8^ The results from our previous study showed that in the normal control group, the fibrinogen levels detected by the Clauss method and PT‐derived assay were 3.56 ± 0.70 and 3.95 ± 0.66 g/L, respectively, indicating a remarkable correlation (*p* > 0.05, *r* = 0.900); in the CD patient group, the fibrinogen levels detected by the Clauss method and PT‐derived assay were 0.62 ± 0.19 and 3.70 ± 0.88 g/L, respectively. The fibrinogen level detected by the Clauss method was significantly lower than that by the PT‐derived assay, indicating a poor correlation (*p* < 0.001, *r* = 0.522). Receiver operating characteristic curve analysis of diagnostic performance indicated that when the fibrinogen antigen/activity ratio (PT‐derived assay result/Clauss method result) was >1.43, both the sensitivity and specificity of CD diagnosis were 100%.^8^ Therefore, fibrinogen detection by the Clauss method and the PT‐derived fibrinogen assay showed similar results in healthy people. However, in patients with CD, the fibrinogen level detected by the PT‐derived fibrinogen assay was significantly higher than that detected by the Clauss method. Fibrinogen detection using a combination of the two methods can be employed for CD diagnosis.

According to existing experimental technologies and literature results, the diagnostic scheme for CD has been proposed as follows^2^, ^9^: (1) Clinical manifestations: Most patients have no symptoms, and several patients present with mild bleeding and/or thrombosis. (2) Coagulation function: The fibrinogen level is in the normal range or increased as detected by the PT‐derived assay and is significantly decreased as detected by the Clauss method; the fibrinogen antigen/activity ratio (PT‐derived assay result/Clauss method result) is >1.43 or fibrinogen‐Clauss method result/PT‐derived assay result is <0.7; thrombin time is prolonged; prothrombin time and activated partial thromboplastin time change unremarkably. (3) Family survey: Inheritance is mostly autosomal dominant, and the patient and one of the parents or other family members have similar coagulation performance and clinical manifestations. (4) Genetic testing: Fibrinogen‐deficient gene testing is necessary for suspected patients or to clarify the correlation between patient gene mutations and clinical manifestations of bleeding and thrombosis.

In this study, the fibrinogen level of the pregnant woman showed a significant reduction by multiple tests conducted at the Souzhu Hospital Affiliated to the M Medical University. After consultation with multidisciplinary experts, the proband was misdiagnosed with hypofibrinogenemia. She also visited a provincial maternal and child hospital for diagnosis and treatment; however, there was no definitive diagnosis. A false reduction in fibrinogen level detected by the Clauss method may lead to a misdiagnosis of hypofibrinogenemia accompanied by abnormal coagulation function in patients with dysfibrinogenemia. When the PT‐derived assay or immune turbidimetry is used to detect fibrinogen levels, the results are often normal or higher, making it prone to a missed diagnosis.^10^ The pregnant woman subsequently visited our hospital for diagnosis and treatment; her coagulation function test results were as follows (Table 1).

Zhou et al.^11^ reported that a patient with CD who was misdiagnosed with hypofibrinogenemia suffered a pulmonary embolism after fibrinogen infusion, which caused serious adverse effects on the patient. Therefore, the treatment of patients with CD must be individualized based on whether there is an individual or family history of bleeding symptoms or thrombotic events in combination with the results of thromboelastography and other examinations. For asymptomatic patients, no special treatment is required during pregnancy or surgery except for close observation and monitoring; patients with bleeding symptoms should be administered fibrinogen or cryoprecipitate infusion; patients with thrombotic events should receive prophylactic treatment with low‐molecular‐weight heparin.

## FUNDING INFORMATION

This work was supported by the National Natural Science Foundation of China [grant numbers 81560342, 2015].

## CONFLICT OF INTEREST

The authors declare that they have no known competing financial interests or personal relationships that could have appeared to influence the work reported in this paper.

## Data Availability

The data that support the findings of this study are available from the corresponding author upon reasonable request.
